# Expert consensus on an in vitro approach to assess pulmonary fibrogenic potential of aerosolized nanomaterials

**DOI:** 10.1007/s00204-016-1717-8

**Published:** 2016-04-27

**Authors:** Amy J. Clippinger, Arti Ahluwalia, David Allen, James C. Bonner, Warren Casey, Vincent Castranova, Raymond M. David, Sabina Halappanavar, Jon A. Hotchkiss, Annie M. Jarabek, Monika Maier, William Polk, Barbara Rothen-Rutishauser, Christie M. Sayes, Phil Sayre, Monita Sharma, Vicki Stone

**Affiliations:** PETA International Science Consortium Ltd., London, UK; University of Pisa, Pisa, Italy; Contractor Supporting the National Toxicology Program Interagency Center for the Evaluation of Alternative Toxicological Methods, Integrated Laboratory Systems, Inc., Research Triangle Park, NC USA; North Carolina State University, Raleigh, NC USA; NTP Interagency Center for the Evaluation of Alternative Toxicological Methods, Raleigh, NC USA; West Virginia University, Morgantown, WV USA; BASF Corporation, Florham Park, NJ USA; Environmental and Radiation Health Sciences Directorate, Health Canada, Ottawa, ON Canada; The Dow Chemical Company, Midland, MI USA; U.S. Environmental Protection Agency, Research Triangle Park, NC USA; Evonik Industries AG, Hanau-Wolfgang, Germany; Adolphe Merkle Institute, University of Fribourg, Fribourg, Switzerland; Department of Environmental Science, Baylor University, Waco, TX USA; nanoRisk Analytics, LLC, Washington, MD USA; Heriot-Watt University, Edinburgh, UK

**Keywords:** Inhalation toxicity, Multi-walled carbon nanotubes, MWCNTs, In vitro testing strategies, Regulatory risk assessment, Pulmonary fibrosis

## Abstract

The increasing use of multi-walled carbon nanotubes (MWCNTs) in consumer products and their potential to induce adverse lung effects following inhalation has lead to much interest in better understanding the hazard associated with these nanomaterials (NMs). While the current regulatory requirement for substances of concern, such as MWCNTs, in many jurisdictions is a 90-day rodent inhalation test, the monetary, ethical, and scientific concerns associated with this test led an international expert group to convene in Washington, DC, USA, to discuss alternative approaches to evaluate the inhalation toxicity of MWCNTs. Pulmonary fibrosis was identified as a key adverse outcome linked to MWCNT exposure, and recommendations were made on the design of an in vitro assay that is predictive of the fibrotic potential of MWCNTs. While fibrosis takes weeks or months to develop in vivo, an in vitro test system may more rapidly predict fibrogenic potential by monitoring pro-fibrotic mediators (e.g., cytokines and growth factors). Therefore, the workshop discussions focused on the necessary specifications related to the development and evaluation of such an in vitro system. Recommendations were made for designing a system using lung-relevant cells co-cultured at the air–liquid interface to assess the pro-fibrogenic potential of aerosolized MWCNTs, while considering human-relevant dosimetry and NM life cycle transformations. The workshop discussions provided the fundamental design components of an air–liquid interface in vitro test system that will be subsequently expanded to the development of an alternative testing strategy to predict pulmonary toxicity and to generate data that will enable effective risk assessment of NMs.

## Introduction

Inhalation is considered an important route of entry into the human body for aerosolized nanomaterials (NMs) released into the environment (during production, processing, intended usage, or disposal of products). Research into the potential hazards of these materials has therefore often focused on effects on the respiratory tract as a target tissue. Traditional in vivo inhalation toxicity tests require large numbers of animals, specialized facilities, and expertise, and are resource-intensive with respect to both time and cost. Concerns related to the use of animals, cost, and time as well as the technical difficulty and weak predictive ability of the 90-day rodent inhalation test for humans have motivated interest in developing in vitro lung cell-based methods. As the prevalence and diversity (different functionalization or physical–chemical properties) of NMs in commerce continues to grow, so does the need for efficient and accurate, human-relevant test methods to assess the potential hazards associated with NMs in a timely manner.

With this in mind, in February 2015, the PETA International Science Consortium Ltd. co-organized with the US National Toxicology Program Interagency Center for the Evaluation of Alternative Toxicological Methods a workshop that was attended by international representatives from industry, government, academia, and non-governmental organizations with expertise in in vivo and in vitro lung systems, respiratory toxicology, inhalation particle dosimetry, nanotoxicology, and hazard and human health risk analysis. The goal of the workshop was to review the state of the science and determine the technical needs to develop an in vitro system that will reduce and eventually replace the use of animals for evaluating the potential inhalation toxicity of NMs in a regulatory setting. The workshop discussion focused specifically on the development and preliminary assessment of the relevance and reliability of an in vitro test system to predict the development of pulmonary fibrosis by assessing pro-fibrogenic markers in lung cells cultured at the air–liquid interface (ALI) following exposure to aerosolized NMs, such as multi-walled carbon nanotubes (MWCNTs). This report provides an overview of the discussions on the design of an in vitro system for the prediction of pulmonary fibrosis.

## Regulatory landscape

The increasing use of NMs in consumer-based products warrants a thorough evaluation of their eco- and biological impacts. The general consensus among various international jurisdictions is that the existing regulatory frameworks are appropriate for the assessment of NMs; however, some adaptations have been made or may be required to consider “nano”-specific properties (OECD 2012a; Scott-Fordsmand et al. 2014; Stone et al. 2014). At this time, the US Environmental Protection Agency (US EPA) Toxic Substances Control Act (TSCA) addresses one of the highest numbers of NM-containing products of any authority (more than 160 NMs reviewed to date) (2015) and, the majority of US EPA Premanufacture notices (PMNs) for NMs are for MWCNTs [for details on US EPA’s New Chemicals Program, see (Godwin et al. 2015)]. The information pertaining to the assessment of NMs by regulatory agencies in the USA, European Union (EU), and Canada is described in Table 1. A description of exposure assessment tools for NMs is available online (Hanes et al. 2000).

**Table 1:** USA, European, and Canadian regulatory frameworks relevant to and specific for nanomaterials

Location	Regulatory body	Regulation	NM-specific inclusions
USA	Food and Drug Administration	Federal Food, Drug, and Cosmetic Act (FD&C Act)	None
	Environmental Protection Agency	Federal Insecticide, Fungicide, and Rodenticide Act (FIFRA)	Significant New Use Rules (SNURs) for any new uses not provided in the original submission
		Toxic Substances Control Act (TSCA)	Premanufacture notice (PMN) required for new NMs
Europe	European Chemicals Agency	Registration, Evaluation, Authorisation and Restriction of Chemicals (REACH)	None
		Classification, Labelling and Packaging (CLP)	None
		Biocidal Products Regulation (BPR)	Products containing NMs are excluded from the simplified authorization procedure. Information regarding the nanoform needs to be submitted separately, and the nanoform is thoroughly assessed for potential risk
Canada	Health Canada and Environment Canada	Canadian Environmental Protection Act, 1999 (CEPA 1999) via New Substances Notification Regulations (NSNR) (Chemicals and Polymers) (SOR/2005-247 2005)	Significant New Activity (SNAc) notices issued for short-tangled MWCNTs

While different jurisdictions vary in regulatory requirements and working definitions for NMs, all require exposure and toxicity data for risk assessment and management purposes. The growing number of NMs in consumer products requires ways to prioritize them for assessment in an efficient and cost-effective manner. Factors, such as production volumes, extent of use in consumer products, and evident bio- or ecological toxicity (based on existing data), can aid in the prioritization process.

## Design of an integrated approach to testing and assessment

Often for aerosolized materials, human risk analysis is based upon 90-day inhalation studies using rodents [e.g., Organisation for Economic Co-operation and Development (OECD) test guideline 413 (OECD 2009)]. While these longer-term rodent tests have been historically used for risk assessment purposes, they do not perfectly predict the human response. There are clear anatomical, physiological, and molecular differences between humans and rodents that impact the deposition, transport, and potential toxicity of inhaled particles. Such differences include the dimensions of the airway architecture and the impact of increased nasal deposition in obligate nose-breathing species, such as rats, compared to humans. Additionally, the 90-day OECD inhalation test guideline does not provide information about the activation of complex signaling pathways.^1^

In vitro test methods provide the opportunity to investigate such mechanisms in detail as well as the potential to be scaled for higher throughput (Nel et al. 2012; Godwin et al. 2015), thus addressing the need to rapidly test large numbers of NMs. However, a single assay is unlikely to be able to accommodate the assessment of the complexity of the full respiratory tissue response to NMs or other substances; instead, a battery of complimentary tests will be required.

**Fig. 1:**
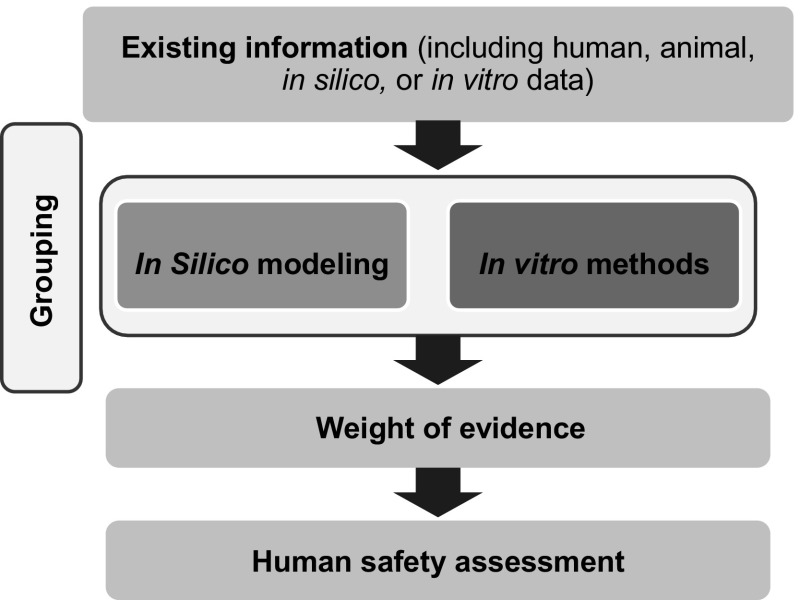
Integrated non-animal approach to assess the inhalation toxicity of aerosolized nanomaterials. This approach should include the use of existing information, grouping (e.g., categorization of NMs based on exposure, mode of action, or physicochemical properties, among other factors), in silico modeling, and the use of in vitro methods of varying complexity

In addition to in vitro assays, a non-animal approach (one that does not use live animals) relevant for assessing inhalation hazard of NMs may include the use of existing data (in vivo or in vitro), grouping (i.e., categorization based on similar attributes or features of interest), and read-across [i.e., predicting the toxicity of one substance based on data from other substance(s)] (Oomen et al. 2014), and in silico (computational) models (Winkler et al. 2012). In vitro testing representing the lung can range from simple cell-free biochemical assays (Zielinski et al. 1999; Mudway et al. 2004), to submerged cell monoculture systems (Foucaud et al. 2007; Rotoli et al. 2008; Foucaud et al. 2010; McCarthy et al. 2011; Banga et al. 2012; Bhattacharya et al. 2013; Nymark et al. 2013; Gliga et al. 2014), through to exposing three-dimensional (3D) cultures grown at the air–liquid interface (ALI) which contain a fully differentiated epithelium with more than one cell type and a morphology similar to that observed in vivo (Klein et al. 2013; Herzog et al. 2014). Each test system can be used to evaluate biomarkers (e.g., antioxidant depletion or pro-inflammatory cytokine expression) linked to pulmonary pathologies (Fig. 1). To be useful for regulatory risk assessment, these biomarkers should include those that address both early and late events in pathologies and diseases, such as fibrosis, chronic obstructive pulmonary disease (COPD), emphysema, and asthma (both sensitization/induction and elicitation/exacerbation. Each of the methods involved in a non-animal approach has advantages and limitations which are important to understand when used for human risk assessment. This workshop report focuses on identifying and recommending the key features that should be considered when developing and evaluating an ALI system for inhalation toxicology. As for other non-animal approaches, development of an ALI system requires benchmark (control) toxic and non-hazardous substances or particles for which considerable human and/or animal data are already available in order to allow comparison of results and to aid in their interpretation. As experience with, and confidence in, the test system increases, its use could be expanded to the toxicity ranking (e.g., fibrotic potential) of substances or particles for use in regulatory assessment.

## Fibrosis induction by MWCNTs: an end point relevant to assessing NM hazard

Fibrosis, in general, is a deregulated wound-healing process and involves uncontrolled deposition of extracellular matrix (e.g., collagen) leading to the formation of scar tissue. In the lower respiratory tract, excessive fibrosis (e.g., after high dose or prolonged exposure to a damaging substance) results in a loss of tissue elasticity and reduced lung function (Palecanda and Kobzik 2001; Oberdorster et al. 2005; OECD 2012b). Fibrosis is of key regulatory importance because it is an important pathological consequence, which in itself is debilitating, but also is often associated with other pathologies, such as particle-induced lung cancers, since both are driven in part by chronic inflammation.

Among the different types of NMs that are commercially available today, carbon nanotubes (CNTs), especially MWCNTs, stand out as model NMs of research interest for inhalation toxicity. MWCNTs are increasingly synthesized and widely used in consumer products, which has lead to much research on these materials (Vance et al. 2015). The high aspect ratio (i.e., long and narrow fiber-like shape) of CNTs together with their potential for persistence in the lower respiratory tract has raised the concern that long-term exposure to CNTs via inhalation may also lead to pathologies analogous to those observed with other fibrous particles (OECD 2009). In fact, pulmonary fibrosis is the most extensively reported adverse outcome for MWCNTs in existing in vivo studies. Further, a chronic inhalation study has linked MWCNTs (Mitsui 7s) with fibrosis in rodents (Fukushima et al., in preparation). Studies have shown that the potential for inhaled MWCNTs to induce fibrosis is influenced by their physicochemical properties (Ma-Hock et al. 2009; Mishra et al. 2012; Sargent et al. 2014; Francis et al. 2015). Fibrosis is therefore an important and relevant end point to consider for the risk assessment of MWCNT.

A range of in vitro methods have been published that assess the pulmonary toxicity of MWCNTs. A number of these in vitro studies have been directly compared with studies in animals. For example, Taylor et al. (2014) have shown that coating MWCNTs with aluminum oxide decreased the production of certain pro-fibrotic markers (osteopontin, interleukin-6, and tumor necrosis factor-α) in THP-1 human monocytic cells in vitro, which correlated with reduced fibrosis observed in mice after 28 days of exposure via oropharyngeal aspiration. Vietti et al. (2013) demonstrated a dose-dependent stimulation of fibroblast proliferation in vitro following exposure to longer MWCNTs (0.7–3 and 0.7–4 µm for NM 400 (Nanocyl, Belgium) and NM 402 (Arkema, France), respectively), but no induction of fibroblast proliferation following exposure to shorter MWCNTs (0.14–0.5 and 0.7 µm for crushed forms of NM 400 and NM 402, respectively) as measured by cell counting, WST-1 assay, and propidium iodide staining. The fibroblast proliferation observed in vitro reflects the results observed in mice exposed to MWCNTs via oropharyngeal aspiration (increased fibroblast proliferation and collagen accumulation observed 60 days post-exposure) (Vietti et al. 2013). Mishra et al. reported increased TGF-β secretion (as determined by ELISA and Western blotting) from human lung epithelial cells (BEAS-2B cells) and human lung fibroblasts (CRL-1490 cells) as well as increased proliferation (as determined by the WST-1 assay) and collagen production (as measured by the Sircol™ Collagen Assay and Western blotting) in human lung fibroblasts (CRL-1490 cells) after treatment with Mitsui 7 MWCNTs (Godwin et al. 2015; Mishra et al. 2015). The Sircol™ Collagen Assay has been used in situ and in vitro for assessing collagen production, as a quantitative marker of fibrosis induction (Wang et al. 2011). Thus, in addition to providing evidence that MWCNTs may cause fibrosis, these studies provide evidence that in vitro models and a range of biological markers can be used in vitro to predict the fibrotic response. These data therefore provide a starting point for identifying useful biomarkers to assess pulmonary fibrogenic potential in vitro, which can be combined with information from other fibrogenic materials such as silica and asbestos, in order to generate a more comprehensive suite of pro-fibrotic biomarkers. Accordingly, focused efforts are required to further develop this existing knowledge using in vitro methods to assess the fibrogenic potential of particles such as MWCNTs.

## An adverse outcome pathway for pulmonary fibrosis

A decade of nanotoxicology research has revealed a range of mechanisms by which NMs can induce toxicity; however, integration of this knowledge to support regulatory decision making has been a challenge (Fedan et al. 2014; Haniu et al. 2014; Hussain et al. 2014; Kim et al. 2014; Dong et al. 2015). The development of well-defined adverse outcome pathways (AOPs)—a conceptual framework that links a molecular initiating event to an adverse outcome at a biological level of organization relevant to risk assessment (Ankley et al. 2010)—can help in (1) systematically organizing existing scientific information concerning NM-induced toxicity; (2) identifying knowledge gaps; (3) informing the design of relevant in vitro predictive assays and testing strategies to help inform risk assessment; and (4) guiding future research priorities. AOPs are gaining acceptance as a way to assist in regulatory decisions which are based on mechanistic understandings and may reduce reliance on in vivo data (with quantitative dosimetry issues considered separately as part of an integrated approach).For instance, Labib et al. (2016) demonstrated the use of existing transcriptomics data to construct a hypothetical AOP that shows the sequential development of fibrotic lesions in the lungs after initial exposure to MWCNTs. Such a transcriptomics/AOP approach may be applied toward hazard ranking of MWCNTs based on their potential to cause fibrosis and aid in regulatory decision making. There are a large number of AOPs currently under review within the OECD.

**Fig. 2:**
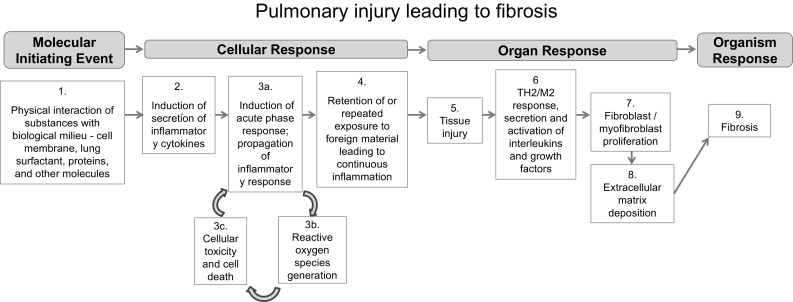
Putative adverse outcome pathway (AOP) for pulmonary fibrosis. The schematic shows the putative molecular initiating event, key events, and adverse outcome of an AOP presented during the workshop

An AOP with a specific molecular initiating event and key events involved in the process of pulmonary fibrosis may inform the selection of in vitro end points and assay design (Fig. 2). Key events to assess can be grouped into low-priority and high-priority categories based on their relevance to the adverse fibrotic outcome and the availability of information and applicable techniques. The lower priority events include initial (pro-) inflammatory signaling (Fig. 2, step 2) assessed by multiplex ELISAs, the formation of reactive oxygen species (ROS; Fig. 2, step 3b), and cellular toxicity and death (Fig. 2, step 3c), which could be determined by assessing ROS generation, epithelial cell apoptosis, and the analysis of mRNA and protein levels of platelet-derived growth factor (PDGF), osteopontin (OPN), tenascin-C (TN-C), and chemokine (C–C motif) ligand 2 (CCL-2). The higher priority key events include fibroblast and myofibroblast proliferation (Fig. 2, step 7), resulting in excessive collagen synthesis and extracellular matrix deposition (Fig. 2, step 8). Thus, AOPs can help identify suitable key events to serve as indicators of fibrotic potential and specific biomarkers to assess using a targeted omics approach. More specifically, addressing the high-priority key events outlined above would include assessing total collagen deposition (e.g., via Sircol™ assay), fibroblast proliferation (via FACS analysis, cell counting under microscope, WST-1 assay, or propidium iodide staining), TGF-β expression (via ELISA or Western blotting for protein levels, or via RT-PCR or Northern blotting for mRNA levels), and overall cytotoxicity (via LDH assay). Integral to the proposed AOP is the bio-persistence of MWCNTs, which when retained in the lung tissue for a long duration after exposure, will contribute to ongoing tissue injury (Fig. 2, step 4) and continued stimulation of wound-healing processes including the secretion of inflammatory mediators and growth factors. This interaction of MWCNTs with lung tissue and the resultant inflammatory response can be qualitatively assessed by several microscopic methods.

## MWCNTs of interest

Ideally, a minimum of two different MWCNTs should be used to develop and test the in vitro system, one that has been demonstrated to be highly pro-fibrogenic and one that induces less fibrosis in in vivo and in vitro studies. Consequently, Mitsui-7s, which have been shown to be highly toxic, and Nanocyl 7000 MWCNTs [NM 400 from the European Commission Joint Research Centre (JRC)], which have been shown to be less toxic, would provide a good comparison (van Berlo et al. 2014). Importantly, both materials have been well characterized and tested for their ability to induce fibrosis in vitro and in vivo (via instillation, pharyngeal aspiration, or nose-only inhalation exposure and assessed up to 90 days post-exposure), and it was demonstrated that the Nanocyl 7000 MWCNTs are fibrogenic but less so than the Mitsui-7s (Ma-Hock et al. 2009; Vietti et al. 2013; van Berlo et al. 2014; Poulsen et al. 2015). In addition, asbestos (e.g., long fiber amosite or crocidolite), which has been shown to induce lung fibrosis in rodents and in humans, could serve as a positive control. A secondary positive control option is crystalline silica (e.g., MIN-U-SIL-5^®^ or DQ12) as it is another relatively insoluble inhaled particle. Negative controls could include a highly soluble refractory ceramic fiber (RCF) (e.g., RCF1) or carbon black (e.g., Printex^®^ 90) (Mast et al. 1994; Bellmann et al. 2001; Sanchez et al. 2011; Hussain et al. 2014) in addition to NM-free air (untreated control for ALI exposures).

## Suggestions for advanced in vitro lung models in nanotoxicology research

There are numerous in vitro lung models that can be used to study the cellular interplay and cellular responses following NM exposure (Jud et al. 2013). They range from simple monocultures (Lehr 2002; Steimer et al. 2005) to highly sophisticated 3D co-culture models (Rothen-Rutishauser et al. 2005; Rothen-Rutishauser et al. 2008; Huh et al. 2010; Klein et al. 2012; Bove et al. 2013; Herzog et al. 2013; Li et al. 2013; Herzog et al. 2014; Chortarea et al. 2015), which represent a more realistic physiological situation (Carterson et al. 2005). While simpler cell culture systems can be useful, with the advantages of being potentially cheaper and more amenable to high-throughput assay design, there have been significant advancements in the development of well-characterized 3D, multi-cellular tissue models that allow for the investigation of cellular interplay between different cell types following exposure to NMs. Furthermore, exposing lung cells cultured at the ALI to aerosolized NMs allows for even more human-relevant exposure conditions.

A number of factors influence the successful use of advanced in vitro models, some of which are outlined here. For any cell culture work, it is critical that details on culture conditions (e.g., dishes, medium, and supplements) are reported, since cell lines can behave differently depending on these variables (Hartung et al. 2002; Coecke et al. 2005). Use of original cell lines obtained from recognized organizations, such as the American Type Culture Collection (ATCC), European Collection of Authenticated Cell Cultures (ECACC), or the German Collection of Microorganisms and Cell Cultures (DSMZ), helps to ensure the authenticity of the cells and to ensure that they have been handled using routine procedures with relatively low passage numbers. Many studies suggest that the use of early passage cells is preferable since later passages of some cells cultures can lose their phenotype or become senescent. Monitoring cellular morphology, structure, and function [e.g., by measuring tight junctions via transepithelial electrical resistance (TEER)] as well as the expression of cell type-specific surface proteins should be used over time to identify whether cells lose their specific phenotype. The use of animal-derived media supplements, such as fetal bovine serum, can pose ethical as well as reproducibility issues (Palecanda and Kobzik 2001; Mishra et al. 2015). Therefore, the use of chemically defined, serum-free medium was recommended, which is also in accordance with the ECVAM Scientific Advisory Committee (ESAC) statement promoting non-animal alternatives to fetal calf serum (ESAC 2008). 3D models can contain a range of different cell types in different orientations and numbers, and to obtain a model best reflecting a normal or diseased state tissue physiology, organ-relevant ratios of cell types should be used and organized in a structure that reflects the tissue of interest.

**Fig. 3:**
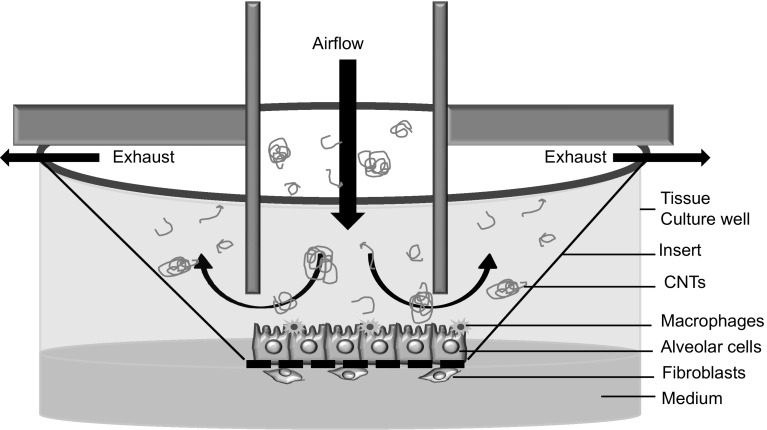
Experimental design of a lung co-culture system to study the fibrotic potential of substances. Schematic shows macrophages and alveolar epithelial cells cultured on a cell culture insert that is exposed at the air–liquid interface to aerosolized MWCNTs, while fibroblasts are cultured on the basal surface of the membrane

Using technologies that are currently available and accessible across laboratories, we recommend an approach which co-cultures fibroblasts on the basal surface of Transwell^®^ or other cell culture inserts with macrophages and alveolar epithelial cells on the apical surface in order to generate an in vitro system to predict fibrosis when exposed to aerosolized NMs at the ALI (Fig. 3). Other co-culture systems have been described which included endothelial cells, dendritic cells, and mast cells (Hermanns et al. 2004; Alfaro-Moreno et al. 2008; Rothen-Rutishauser et al. 2008; Klein et al. 2013); however, because increasing the number of cell types makes it more difficult to maintain optimal culture conditions, only the cells which are central for a pathological process should be prioritized. In the case of a fibrosis model, these are epithelial cells, macrophages, and fibroblasts.

The use of primary cells is optimal, recognizing that the use of primary cells may lead to increased interlaboratory variability. The use of human cells is ideal because the regulatory need is to predict the human, rather than the rodent, response. The use of human cells is also in line with a twenty-first century vision of toxicology (NRC 2007). However, when validating against the historical 90-day rodent inhalation studies, it is unknown whether rodent cells may show greater concordance. There was discussion of using the commercially available reconstructed human lower respiratory tract tissue model, EpiAlveolar™ (MatTek Corp., Ashland, MA) (Jackson et al. 2013); however, insufficient published data are currently available to fully understand its applicability to these studies.

In a static ALI system, factors secreted from the alveolar cells and macrophages are small enough to pass through the pores of the cell culture inserts, but the MWCNT agglomerates may be too large to do so. Thus, this in vitro test system is not ideal for studying the translocation of MWCNTs to the fibroblasts cultured on the basal side. However, the effect of MWCNTs on fibroblasts can be assessed by conducting parallel testing in a simple submerged cell culture system.

## In vitro air–liquid interface exposure systems

In order to more closely mimic the conditions of the human lung, to maximize the human relevance of in vitro testing, and to simplify the dose metric calculations, the exposure of cells cultured at the ALI to aerosolized materials is of significant research interest. The typical setup of an exposure system built to deliver NMs to ALI cultures involves three components: (1) an aerosol generator, (2) connections and peripherals, and (3) the exposure chamber. The generator creates the aerosol by a technology appropriate to the physical state of the NM. Dust feeding, brush feeding, turntable, acoustic, cyclone sieve, fluidized bed, and string technology have all been used to generate aerosols from powders/dusts, while electrospray and nebulizing generators have been used to aerosolize particles in liquid droplets (Cheng 1986; Moss et al. 1994; Jarabek et al. 2005; Teeguarden et al. 2007; Bouwmeester et al. 2011). The connections and peripherals are the intermediate devices and tubing or channels used to transport, dilute, characterize, and condition the aerosol prior to deposition. The exposure chamber is made of an inert material designed to facilitate the deposition of the aerosol onto the cells without compromising their viability.

A number of commercial in vitro exposure technologies exist which are (1) capable of consistently delivering and depositing aerosols at multiple dilutions, (2) compatible with different types of aerosol generators, (3) capable of determining the deposited dose, (4) compatible with cell culture inserts used for ALI-grown cells, and (5) readily cleaned. However, some desirable traits are found absent in all current technologies, namely that high throughput is not available, and only short-duration (<6 h) single-exposure experiments have been demonstrated for each technology. An overview of aerosol generators and exposure chambers with regard to their applicability to the assessment of MWCNTs is discussed in the sections below.

### Aerosol generation

Each aerosol generation method has advantages and disadvantages. For example, dry aerosol generation more closely mimics the realistic human exposure that would be of regulatory concern. For the dry exposure, the atmosphere around the cell system needs to be humidified to simulate physiological conditions. Wet aerosol generation, on the other hand, involves administration of NMs encased in liquid droplets, which may induce agglomeration and introduce liquid onto the cells, possibly altering the cellular outcome. However, one study has demonstrated that the increase in the volume of the liquid layer does not alter the biological effect of the aerosolized NM (Lenz et al. 2009). Although dry aerosol generation of MWCNTs is preferred over wet in order to link dosimetry considerations to biological outcomes (e.g., fibrosis), the wet generation methods can be used to develop the cell system to predict fibrosis and might provide the advantages of low cost, ease of use, and inter- and intra-laboratory transferability.

To maintain optimal conditions while the cells are inside the aerosol chamber for short exposure (minutes to 1 h), a thermostat-controlled incubation chamber (37 °C) should be used and the exposure system should be humidified to 80–95 % relative humidity. For longer exposures, it is recommended to use HEPES-buffered cell culture media (constant pH) or to supply 5–7 % carbon dioxide for media that are buffered by this gas.

All of the aerosol generation methods discussed during the workshop have been used to aerosolize CNTs; thus, the choice of the appropriate method largely depends on CNT concentration range and the aerosol particle size range desired. For humans, respirable particles (i.e., particles that enter the lower respiratory tract) have a mass median aerodynamic diameter (MMAD) of less than 5 µm while, for rats, respirable particles have a MMAD of less than 2.5 µm. The size of the aerosol particles (individual nanofibers, agglomerates, or aggregates) and the mass deposited on the cells (dose) could potentially lead to differential toxicity outcomes. In addition to the ability to control and determine relevant dose metrics for the end points of interest, the price, ease of cleaning, ease of use, size of the generators, and interlaboratory transferability should be considered when evaluating equipment for such studies.

### Exposure chamber design

Factors to consider when selecting an ALI exposure chamber include reproducibility, ease of cleaning and use, cost, commercial availability, compatibility with test materials, number and size of the wells in the chamber, configuration, modularity, and flexibility to add online and off-line equipment. Many of the chambers share common features, such as their compatibility with commercially available cell culture inserts, a mechanism to regulate temperature during exposure, and their ability to be used with multiple aerosol generators and to expose cells cultured at the ALI to aerosolized MWCNTs. However, there are differences that make each system unique. For example, the available ALI exposure chambers vary in the flexibility of connections and peripherals. While modularity can allow for more sophisticated testing, it also adds a level of complexity, thereby requiring more expertise and training for use relative to simpler systems. It is important to note that, for modular systems, small alterations to the connections and peripherals (such as a change in the length of the tubing used) can alter results.

### Dosimetry

Accurate and appropriate administration and monitoring of NM deposition is critical, with aspects such as the relevant target scenarios for administered doses and cellular dose metrics to use, modes of NM deposition, and measurement of administered versus cellular dose to be included in considerations. The administered dose range should include concentrations relevant to occupational exposures to CNTs, encompassing several deposited/cellular doses in order to obtain a dose–response curve. For carbon nanotubes and carbon nanofibers, the National Institute for Occupational Safety and Health (NIOSH) recommends that exposures be controlled to less than 1 μg/m^3^ of respirable elemental carbon as an 8-hr time-weighted average^2^ and that, until exposure limits are established, to follow best practices to reduce exposures (e.g., engineering controls, personal protective equipment, and medical screening or surveillance) (NIOSH 2013). While this recommended exposure limit is helpful for regulatory purposes, it has not been adopted as a permissible exposure limit by the Occupational Safety and Health Administration and is based on limited data (NIOSH 2013). Therefore, in addition to testing concentrations that can be related to the occupational exposures of CNT, a dose range that represents doses shown to cause fibrosis or indicate pro-fibrotic potential in existing in vivo and in vitro studies should also be tested (see also dosimetry modeling below) (Li et al. 2013; Godwin et al. 2015).

There are challenges associated with the administration of a dose in a consistent and reproducible manner to cells cultured at the ALI. The gravitational settling of MWCNTs takes a long time due to their low density and mass, which may lead to issues keeping the cell layer moist at the ALI. However, most of the available exposure systems are capable of maintaining constant temperature and humidity for the duration of exposure. Regardless, the cell system should be monitored throughout the exposure using TEER measurements and histopathological analysis at different time intervals to evaluate epithelial cell barrier integrity. Deposition could be enhanced using electrostatic force, but there are significant concerns regarding the applicability of this method to realistic exposures.

Mass, surface area, and nanostructure number should be considered as dose metrics because all three are considered at the OECD level (OECD 2012b). In fact, deposition of MWCNTs can be measured as a function of mass [quartz crystal microbalance (QCM)], surface area, or particle count (electron microscopy grid). ALI systems that are compatible with a QCM sensor for monitoring deposited dose are preferred because they can measure deposition in the nanogram range. While this is simple for a dry aerosol, it is more difficult for a wet aerosol as discussed above. Additional measurement of MWCNTs on a count basis may enable hazard characterization on a fiber count basis analogous to asbestos.

## Characterization of dose at multiple stages during NM life cycle

**Fig. 4:**
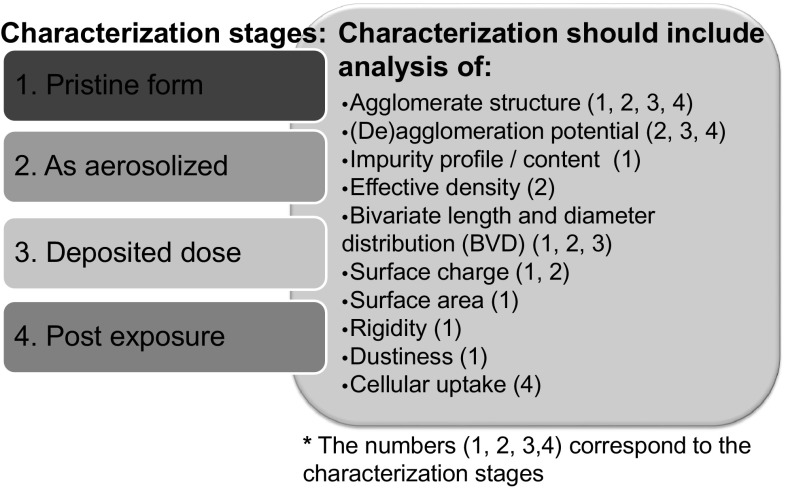
NMs characterization during the course of a study. Ideally, NMs will be characterized in their original dry form, as aerosolized, as deposited, and at multiple time points during the course of the study. The characterization conducted will depend on the stage of the NM [i.e., (*1*) pristine form; (*2*) as aerosolized; (*3*) deposited dose; and (*4*) post-exposure] and, in this figure, the numbers *1* through *4* in parentheses following each parameter correspond to the four stages of NMs listed

When developing an in vitro test system to gauge potential hazards related to respiratory toxicology, characterizing the aerosol delivery to cells is critical for accurate interpretation of the results. The dosimetry determinants that dictate aerodynamics and deposition mechanisms (such as density and the bivariate (length and width) size distribution), the amount of NMs that reach the cell surface (deposited dose), and the amount of NMs taken up by the cells (cellular dose) are all critical parameters needed to interpret the observed biological responses (Oberdorster et al. 2005; Teeguarden et al. 2007). Additionally, NM life cycle transformations, including agglomeration (either homoagglomeration or heteroagglomeration with other colloids), dissolution, and changes in surface properties (biodegradation and corona formation), should be well characterized during the course of the assay in an effort to take into account specific transformations that may occur, and to support dose translation across experimental systems (i.e., in non-animal vs. in vivo systems) as well as data integration for environmental conditions for real-world applications. Figure 4 outlines considerations such as when to characterize the NM, what parameters to monitor, and what techniques to use for characterization.

When NMs are purchased, information about the NM generation method, the date of manufacture, and how the material is stored and supplied should be obtained from the supplier and reported in publications. While not always achievable due to budget and resources, it is ideal to characterize NMs (1) in their original dry form; (2) as aerosolized particles; (3) as deposited on the cell surface; and (4) following cellular uptake at multiple post-exposure time points throughout the course of the study. For the purposes of the case study discussed during the workshop, characterization data on the pristine form of the Mitsui-7 MWCNTs has already been reported in the literature (Poland et al. 2008; Taquahashi et al. 2013; Sargent et al. 2014). Additionally, characterization information for standard reference materials, such as the Nanocyl 7000, is available through the Joint Research Center (JRC 2014) and has also been reported in the literature (Vietti et al. 2013). It would be beneficial to coordinate additional efforts like the Minimal Information for Nanomaterial Characterization (MINChar) Initiative to develop a minimal list of recommended characterization parameters for NM studies (Bouwmeester et al. 2011).

Aerosolized NMs can be characterized by an aerodynamic particle sizer (APS), scanning mobility particle sizer (SMPS) spectrometer, and micro-orifice uniform deposit impactor (MOUDI). It is crucial to characterize aerosolized NMs to determine the form that the cell systems are being exposed to in order to extrapolate from system to system (DeLoid et al. 2014). In other words, aerodynamic properties and aerosol behavior of MWCNTs in their singlet form may be very different from the “tumbleweed” form that occurs following NM aerosolization. While the density of the singlet form has traditionally been used for most materials, the density of “as-generated” structures is more important for MWCNTs. In the “tumbleweed” form, the aerodynamic diameter and the effective density of the MWCNTs are lower, which is why larger particles can be inhaled.

Deposited structures can be characterized qualitatively using microscopy (e.g., CytoViva^®^), and deposited mass quantitatively determined using a QCM. Particle count and size information can be determined using electron microscopy. The cellular internalization and possible localization of NMs can be examined qualitatively by TEM or quantitatively by inductively coupled plasma mass spectrometry (ICP-MS), which measures the amount of trace metal impurities associated with the MWCNTs.

Ideally, characterization of the original NM should include analysis of properties, such as surface area [using the Brunauer, Emmett, and Teller (BET) method], dustiness (according to DIN 55992-2), impurity profile/content, effective density (using gas pycnometry), rigidity (via imaging and field emission SEM), and bivariate distribution (BVD) (i.e., length and diameter) (Lehman et al. 2011). The properties that should be characterized during NM exposure are agglomerate structure, (de)agglomeration potential (via the Tendel test), and surface charge (when NMs are in a relevant medium). The aforementioned characteristics dictate the life cycle transformations that NMs undergo and therefore their potential ecological and biological effects. For instance, the rigidity and length of MWCNTs impact the level of agglomeration, which in turn dictates the localization and toxicological impact of nanotubes in the physiological system (Donaldson et al. 2010; Klein et al. 2012).

## Dosimetry modeling to aid experimental design, evidence integration, and inferences

Dosimetry modeling is well established as a useful bridge to link exposure to internal dose and the resultant response. Calculation of the internal dose associated with exposure improves the accuracy of risk assessment (Jarabek et al. 2005). Dosimetry models quantitatively describe the aerodynamics of inhaled aerosols, including inhalability and mechanisms of deposition (interception, impaction, sedimentation, and diffusion), in the respiratory tract to predict deposited dose. Dosimetry models also quantitatively describe clearance kinetics (including mechanisms of mucociliary clearance, translocation, and dissolution) to estimate the dose retained in respiratory tract tissues (retained dose is equal to inhalability plus deposition, minus clearance). The relative contribution of impaction, interception, sedimentation, and diffusion to deposition is dependent on the respiratory tract region and species-specific parameters (Asgharian et al. 2014). Species-specific airway anatomy and physiology, such as ventilation rate and breathing mode (i.e., nasal, oronasal, or mouth), are critical determinants of particle and fiber aerodynamics, and vary with age and exertion level or activity pattern (e.g., resting vs. exercise). Particle parameters including density and size distribution are also critical determinants of aerodynamics, and biopersistence determines physical dissolution rates involved in clearance kinetics. Additionally, to properly describe fiber aerodynamics, the bivariate distribution of length and diameter is required (Cheng 1986; Moss et al. 1994; Vincent 2005).

Dosimetry models, such as the multi-path particle dosimetry (MPPD) model (available from Applied Research Associates (ARA), Inc.), have been used (1) as the basis of size-selective exposure sampling and the National Ambient Air Quality Standard (NAAQS) for particulate matter; (2) for interspecies extrapolation of particle exposures in various species; (3) to understand uncertainties when using exposure as the dose metric; and (4) to provide insights into human variability due to age, ventilation rate, or disease (Anjilvel and Asgharian 1995; RIVM 2002). ARA, Inc. has released a version of the MPPD model developed in collaboration with NIOSH that describes deposition and clearance kinetics for NM to predict retained dose, and an US EPA collaboration is developing an extension of MPPD to model asbestos fibers. Furthermore, emphasis on the need to describe dosimetry of in vitro test systems for accurate in vitro to in vivo extrapolation (IVIVE) motivated the development of the in vitro sedimentation, diffusion, and dosimetry (ISDD) model to predict particles delivered in solution to submerged monolayer cells and to understand the kinetics of NMs in in vitro systems (Hinderliter et al. 2010).

Incorporating principles of dosimetry and quantitatively addressing fiber kinetics with in silico models will increase the predictive power of the test system and its usefulness in risk assessment (Stöber 1972; Anjilvel and Asgharian 1995; Sturm and Hofmann 2006; Sturm 2009, 2011; Sturm and Hofmann 2009). *In silico* modeling approaches can be combined with existing data to devise a dosimetry paradigm for the design and interpretation of in vitro studies. The application of such modeling as an approach to improve the experimental design of in vitro test systems was discussed at the workshop. The relevant human scenario for regulation should be defined (e.g., exposure concentration, duration, and population) to aid the experimental design and frame the inference approach (Holsapple et al. 2005; Jarabek et al. 2005; Teeguarden et al. 2007; Gangwal et al. 2011). Experimental parameters (including test species, exposure concentration, duration, and regiment) from existing studies can be used to describe internal dose in the in vivo toxicity studies and support IVIVE. Potential dose metrics should be based on the likely mode of action for NMs (Jarabek et al. 2005; Oberdorster et al. 2005; Teeguarden et al. 2007). Ideally, relevant parameters for the construction of the dose metrics are measured in the test systems. These parameters include deposited mass, number, and surface area of the NM in the alveolar region normalized to species-specific factors, such as alveolar surface area (NIOSH 2013). Reference values can be used for species- and strain-specific parameters. Additionally, in silico models can use existing exposure data from animal tests to predict an equivalent exposure in vitro.

## Conclusions and recommendations

**Fig. 5:**
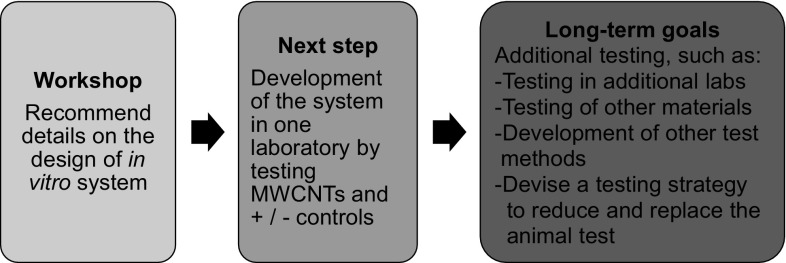
Project plan. The ultimate goal was to devise a human-relevant replacement for the 90-day rodent inhalation test for the hazard identification of inhaled NMs. This is a long-term project that will involve an integrated battery of multiple assays; however, the initial study discussed during this workshop will be a proof of concept to determine whether a tiered testing approach for the assessment of fibrogenic pathways can be developed to provide confidence in prioritization decisions in the near-term. During the process outlined, it would be ideal to share study protocols and data with regulators in order to facilitate regulatory acceptance of the method

This workshop discussed multiple components of a potential predictive in vitro test system for the determination of NM fibrogenic potential (Fig. 5). Work is currently underway to implement the workshop recommendations into the development of such a system. The goal was to develop an in vitro system that can contribute to addressing the regulatory safety testing requirements for inhaled NMs, thereby reducing time and cost of testing as well as decreasing the number of animals used for this purpose. The ultimate goal was to incorporate this in vitro fibrosis system into an integrated approach for assessing the toxicity of inhaled NMs that can replace the subchronic rodent inhalation assay and protect human health.

The first step in implementing the workshop recommendations for developing the in vitro pulmonary fibrosis system is the establishment of a robust, reproducible co-culture system containing epithelial cells, macrophages, and fibroblasts. The second step is the implementation and optimization of an aerosol generation system along with characterization techniques that can generate a reliable aerosol of MWCNTs and the control particles. The third task involves selection of a target exposure scenario to aid experimental design and provide inference structure for IVIVE. The target scenario includes determining a range of concentrations that bracket concentrations used in historical in vivo tests and measuring needed parameters to describe dose metrics. The fibrotic response can be assessed following exposure to a range of doses for positive and negative benchmark particles and then to the MWCNTs. Standard protocols, such as those for cell culture conditions (e.g., cell seeding densities, medium conditions, and different co-cultures), aerosol generation (e.g., air flow and method of generation), and in silico modeling (list of NM properties and parameters linked to respiratory system) will need to be developed for all aspects of the system to facilitate interlaboratory transferability. In addition to testing co-cultures in an ALI system, simple mono- or co-culture submerged cell systems could be tested in parallel to see whether comparable results are obtained.

After the system has been optimized in one laboratory, testing should be expanded to additional laboratories to show interlaboratory reproducibility in order to establish whether such a system could be more widely used for predicting human health effects. The MWCNTs suggested (Mitsui-7s and Nanocyl 7000 MWCNTs) have already been tested in various in vitro and in vivo studies, including 90-day rodent inhalation studies; thus, it will be possible to examine the validity of the in vitro test system using available data.

**Fig. 6:**
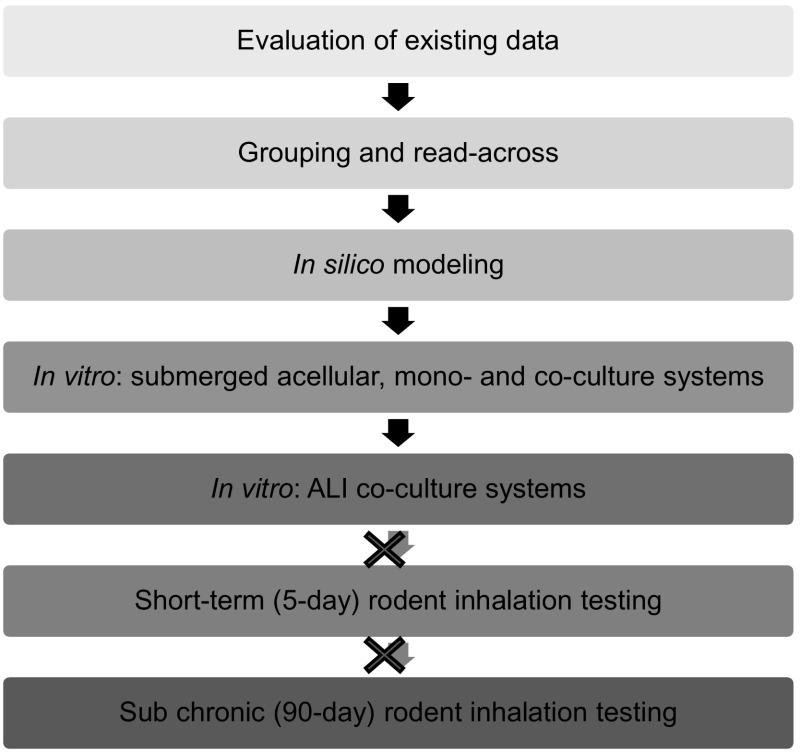
Proposed tiered testing approach for the assessment of the inhalation toxicity of nanomaterials. As confidence grows in the non-animal methods, the goal is to replace the high cost, slow, and technically difficult animal inhalation studies

The study described would be a proof of concept to determine whether a tiered testing approach for the assessment of (pro-)fibrogenic pathways can be developed to provide confidence in prioritizing decisions (Fig. 6). The full data set and protocols from the testing conducted to develop the ALI system could be made available to regulatory agencies for consideration. While the method is undergoing consideration for regulatory use, a near-term use of the system could be for manufacturers to apply it in a safety-by-design approach; for example, the in vitro testing system can be used in conjunction with predictive cheminformatics and read-across approaches during the early stages of materials design and production to decide which NMs will be included in product development. Also in the near-term before the method may be generally accepted for regulatory use, companies may conduct the in vitro method alongside the required 90-day inhalation test using the same materials and share the results with regulators so that both the companies and regulators can assess the similarities and differences in the prediction of NM fibrogenic potential of the same test material using the two (in vitro/in vivo) testing schemes.

Longer-term plans could include using the system to test other NMs and non-nanosubstances and to rank substances in the order of toxicity. Ultimately, the development of OECD and US EPA test guidelines will be important if the method is to be widely used in a regulatory context. To be incorporated into test guidelines, the system would need have demonstrated relevance and reliability to adverse lung effects considered in regulatory contexts (OECD 2005).

While most of the workshop discussions focused on an achievable first-generation in vitro test system, there was also discussion of the ways in which the test system could be expanded to further mimic the human situation. For example, rather than manually seeding cells, bioprinting could more reproducibly assemble thin layers of cells (Horvath et al. 2015). Additionally, primary cells isolated from diseased patients could be used to mimic human disease states. Furthermore, fluidic models and “human-on-a-chip” technologies could be used to allow for complex interactions between different tissue types. Examples of such models include one developed during the European Commission’s 7th Framework Programme-sponsored InLiveTox project (InLiveTox 2012), the lung-on-a-chip model developed at Harvard University’s Wyss Institute (Huh et al. 2010), the captive bubble surfactometer system (Schurch et al. 2014), and the moving membrane air–liquid interface (MALI) bioreactor system developed at the University of Pisa, Italy (Cei et al. 2014). Overall, a number of options exist to transform a static model to include fluidics and membrane mobility. The developed system could also be modified to examine end points other than fibrosis, such as COPD, emphysema, or asthma.

## Abbreviations

ALI: Air–liquid interface
AOP: Adverse outcome pathway
APS: Aerodynamic particle sizer
ARA: Applied Research Associates, Inc
ATCC: American Type Culture Collection
BET: Brunauer, Emmett, and Teller
BPR: Biocidal Products Regulation
BVD: Bivariate distribution
CCL-2: Chemokine (C–C motif) ligand 2
CEPA: Canadian Environmental Protection Act
CLP: Classification, Labelling and Packaging
CNT: Carbon nanotubes
COPD: Chronic obstructive pulmonary disease
DSMZ: German Collection of Microorganisms and Cell Cultures
ELISA: Enzyme-linked immunosorbent assay
EPA: Environmental Protection Agency
ESAC: ECVAM Scientific Advisory Committee
FACS: Fluorescence-activated cell sorting
ICP-MS: Inductively coupled plasma mass spectrometry
ISDD: In vitro sedimentation, diffusion, and dosimetry
IVIVE: In vitro to in vivo extrapolation
JRC: Joint Research Centre
LDH: Lactate dehydrogenase
MALI: Membrane air–liquid interface
MMAD: Mass median aerodynamic diameter
MOUDI: Micro-orifice uniform deposit impactor
MPPD: Multiple-path particle dosimetry
MWCNT: Multi-walled carbon nanotube
NAAQS: National Ambient Air Quality Standards
NGO: Non-governmental organization
NIOSH: National Institute for Occupational Safety and Health
NM: Nanomaterial
NSNR: New Substances Notification Regulations
OECD: Organisation for Economic Co-operation and Development
OPN: Osteopontin
PDGF: Platelet-derived growth factor
PMN: Premanufacture notice
QCM: Quartz crystal microbalance
RCF: Refractory ceramic fiber
REACH: Registration, Evaluation, Authorisation and Restriction of Chemicals
SEM: Scanning electron microscope
SMPS: Scanning mobility particle sizer
SNAc: Significant New Activity
SNURs: Significant New Use Rules
TEER: Transepithelial electrical resistance
TGF-β: Transforming growth factor beta
TN-C: Tenascin C
TSCA: Toxic Substances Control Act

## Footnotes

1 OECD test guideline 413 states that it was designed to characterize subchronic toxicity by the inhalation route and to provide data for quantitative inhalation risk assessment. In this test, 10 male and 10 female rodents are exposed 6 h per day, 5 or 7 days per week, during a 90-day period to at least three concentration levels, filtered air (negative control), and/or the vehicle (vehicle control). The results of the study include hematology, clinical chemistry, ophthalmology, gross pathology, organ weights, and histopathology. For more details, see OECD (2009). Test No. 413: Subchronic Inhalation Toxicity: 90-day Study, OECD Publishing.

2 Few occupational exposure limits exist specifically for nanomaterials. Certain nanoparticles may be more hazardous than larger particles of the same substance. Therefore, existing occupational exposure limits for a substance may not provide adequate protection from nanoparticles of that substance.
